# Characterizing domain-specific open educational resources by linking ISCB Communities of Special Interest to Wikipedia

**DOI:** 10.1093/bioinformatics/btac236

**Published:** 2022-06-27

**Authors:** Alastair M Kilpatrick, Farzana Rahman, Audra Anjum, Sayane Shome, K M Salim Andalib, Shrabonti Banik, Sanjana F Chowdhury, Peter Coombe, Yesid Cuesta Astroz, J Maxwell Douglas, Pradeep Eranti, Aleyna D Kiran, Sachendra Kumar, Hyeri Lim, Valentina Lorenzi, Tiago Lubiana, Sakib Mahmud, Rafael Puche, Agnieszka Rybarczyk, Syed Muktadir Al Sium, David Twesigomwe, Tomasz Zok, Christine A Orengo, Iddo Friedberg, Janet F Kelso, Lonnie Welch

**Affiliations:** Centre for Regenerative Medicine, University of Edinburgh, Edinburgh, UK; Faculty of Science, Engineering and Computing, Kingston University, London, UK; Faculty of Computing, Engineering and Science, University of South Wales, Pontypridd, UK; Office of Instructional Innovation, Ohio University, Athens, OH, USA; Department of Anesthesiology, Perioperative and Pain Medicine, Stanford School of Medicine, Stanford University, Stanford, CA, USA; ISCB Regional Student Group Bangladesh, Dhaka, Bangladesh; Faculty of Veterinary, Animal and Biomedical Sciences, Sylhet Agricultural University, Sylhet, Bangladesh; BCSIR Dhaka Laboratory, Bangladesh Council of Scientific and Industrial Research (BCSIR), Dhaka, Bangladesh; Wikipedia Volunteer, San Francisco, CA, USA; Colombian Institute of Tropical Medicine, CES University, Medellín, Colombia; Department of Molecular Oncology, BC Cancer Agency, Vancouver, BC, Canada; UMRS-1124, INSERM, Université de Paris, Paris, France; Department of Bioengineering, Ege University, Bornova, Turkey; IISc Mathematics Initiative, Indian Institute of Science, Bengaluru, India; Department of Biomedical Data Intelligence, Graduate School of Medicine, Kyoto University, Kyoto, Japan; Wellcome Sanger Institute, Hinxton, Cambridge, UK; European Bioinformatics Institute (EMBL-EBI), Hinxton, UK; School of Pharmaceutical Sciences, University of São Paulo, São Paulo, Brazil; Ronin Institute, Montclair, NJ, USA; Biotechnology and Genetic Engineering Discipline, Khulna University, Khulna, Bangladesh; Genetics and Forensic Studies Unit, Venezuelan Institute of Scientific Research (IVIC), Caracas, Venezuela; Institute of Computing Science, Poznan University of Technology, Poznan, Poland; BCSIR Dhaka Laboratory, Bangladesh Council of Scientific and Industrial Research (BCSIR), Dhaka, Bangladesh; Sydney Brenner Institute for Molecular Bioscience, University of the Witwatersrand, Johannesburg, South Africa; Division of Human Genetics, National Health Laboratory Service and School of Pathology, Faculty of Health Sciences, University of the Witwatersrand, Johannesburg, South Africa; Institute of Computing Science, Poznan University of Technology, Poznan, Poland; Institute of Structural and Molecular Biology, University College London, London, UK; Program in Bioinformatics and Computational Biology, Iowa State University, Ames, IA, USA; Department of Veterinary Microbiology and Preventive Medicine, Iowa State University, Ames, IA, USA; Max Planck Institute for Evolutionary Anthropology, Leipzig, Germany; School of Electrical Engineering and Computer Science, Ohio University, Athens, OH, USA

## Abstract

**Motivation:**

Wikipedia is one of the most important channels for the public communication of science and is frequently accessed as an educational resource in computational biology. Joint efforts between the International Society for Computational Biology (ISCB) and the Computational Biology taskforce of WikiProject Molecular Biology (a group of expert Wikipedia editors) have considerably improved computational biology representation on Wikipedia in recent years. However, there is still an urgent need for further improvement in quality, especially when compared to related scientific fields such as genetics and medicine. Facilitating involvement of members from ISCB Communities of Special Interest (COSIs) would improve a vital open education resource in computational biology, additionally allowing COSIs to provide a quality educational resource highly specific to their subfield.

**Results:**

We generate a list of around 1500 English Wikipedia articles relating to computational biology and describe the development of a binary COSI-Article matrix, linking COSIs to relevant articles and thereby defining domain-specific open educational resources. Our analysis of the COSI-Article matrix data provides a quantitative assessment of computational biology representation on Wikipedia against other fields and at a COSI-specific level. Furthermore, we conducted similarity analysis and subsequent clustering of COSI-Article data to provide insight into potential relationships between COSIs. Finally, based on our analysis, we suggest courses of action to improve the quality of computational biology representation on Wikipedia.

## 1 Introduction

For some years, Wikipedia has been regarded as the most important channel for the public communication of science (Jemielniak and Aibar, 2016) and is frequently accessed as an educational resource in computational biology, with the English-language articles on Bioinformatics and CRISPR being viewed 357 000 and 1.08 million times in 2020, respectively. As an open education resource (OER), offering no-cost access, use, adaptation and redistribution with no or limited restrictions (Miao *et al.*, 2016), Wikipedia provides a free, accessible and immediate alternative to expensive textbooks and paywalled academic journals. OERs have steadily gained traction among educators in the last two decades, and while their perceived value in academic settings may still prompt scepticism due to concerns over quality (Konieczny, 2016), there is evidence that students find OERs to be as good or better than traditional courseware (Abramovich and McBride, 2018). As an encyclopedia, Wikipedia is an ideal first step for learners getting acquainted with new topics. Given that Wikipedia is one of the largest hubs for OERs in the world, it is natural that professional organizations in academic disciplines would rally around the generation and continued maintenance of high-quality articles representing the breadth and depth of their field’s collective body of knowledge.

WikiProjects are groups of Wikipedia editors with a common aim to improve Wikipedia in a specific field of knowledge. Interactions between WikiProjects and external organizations are particularly important in academic fields, where WikiProject members are often domain experts. Founded in 2004, WikiProject Medicine was one of the first WikiProjects (Shafee *et al.*, 2017b) and remains active to this day through partnerships with dozens of medical institutes and universities that have improved Wikipedia’s medical content, in addition to increasing public awareness of their partners via Wikipedia’s readership (Shafee *et al.*, 2017b). A 2011 editorial in *The BMJ* asserted that WikiProject Medicine could become a trusted resource ‘if it was assisted, not shunned’ (Trevena, 2011). By 2013, Wikipedia’s medical content was being viewed more than 6 billion times a year, more than WebMD, the Mayo Clinic and the World Health Organization websites combined (James, 2016).

WikiProject RNA, founded in 2007, created hundreds of articles describing families of non-coding RNAs based on entries in the Rfam database and worked with the Rfam team to redistribute the Wikipedia content as the primary textual annotation of its RNA families (Daub *et al.*, 2008). A follow-up evaluation of this community annotation method noted a dramatic improvement in the content of the Rfam database. By 2011, a significant proportion of web traffic to the Rfam site was found to come from Wikipedia, reflecting its position as an important educational resource (Gardner *et al.*, 2011).

The Computational Biology taskforce of WikiProject Molecular Biology (formerly WikiProject Computational Biology) is an international community of editors, formed in 2007 to organize and improve the now roughly 1500 Wikipedia articles relating to all aspects of computational biology and bioinformatics (O’Neill *et al.*, 2017). Many of these editors are academic or industrial scientists with some expertise in computational biology, who provide important links between their particular specialism, Wikipedia and any professional organizations of which they are a member.

Since 2014, the International Society for Computational Biology (ISCB) has been home to self-organizing COSIs (Communities of Special Interest) (https://www.iscb.org/cms_addon/cosi_reporting_system/COSIs/). COSIs aim to foster a collaborative community focused on specific areas of computational biology, sharing information and expertise. Several of these COSIs (as of 2021, totaling 21) originated from Special Interest Group meetings at the ISCB’s flagship conference, Intelligent Systems of Molecular Biology (ISMB), and now develop their own community events. Both the ISCB and the Wikimedia Foundation, the non-profit organization which operates Wikipedia, share a common core principle of promoting and developing open access to (scientific) information. However, while ISCB conferences have previously hosted several Wikipedia-based tutorials and editathons (O’Neill *et al.*, 2017), the potential benefits of linking ISCB COSIs to Wikipedia have so far not been fully realized.

Making connections between ISCB COSIs and Wikipedia articles relating to those COSIs has benefits for all parties. For COSIs, formalizing lists of relevant articles would allow each community to point to an OER highly specific to their domain. As some overlap in relevant articles among COSIs would be expected, this could help identify potential synergies among COSIs, and potential links with other professional societies. For the ISCB, these lists could help identify COSIs which are not well-represented in publicly accessible information sources. Encouraging COSI members to improve Wikipedia articles in which they are experts would make a quantifiable improvement to the representation of the ISCB’s field online; a long-held view suggests that for academics, improving relevant Wikipedia articles represents a professional responsibility (Callis *et al.*, 2009) and effective communication of bioinformatics with a range of audiences has been identified as a core competency for those in bioinformatics roles (Mulder *et al.*, 2018). Wikipedia would also benefit, through gaining a new community of editors who are highly skilled and have some personal investment in ensuring topics pertaining to their (sub)field are both visible and well-written. Further, the dissemination of current and high-quality domain-specific articles to the public may have an immediate impact on the field’s growth. Recent evidence shows that scientific contributions to Wikipedia can have a causal impact on future developments on the respective field, further emphasizing the unique opportunity Wikipedia affords (Thompson and Hanley, 2017).

The primary goal of this study is to take a first step toward capitalizing on this unique opportunity by linking Wikipedia articles to their relevant ISCB COSIs (and thus to experts with the specialist knowledge to improve these articles) through the creation of a COSI-Article matrix. We also aim to provide analysis of this dataset to provide insight into the variation in computational biology representation on Wikipedia at a domain-specific level. The outcomes of this study will contribute to our longer-term goal of making Wikipedia a high-quality educational resource for computational biology and bioinformatics.

## 2 Materials and methods

In this study, we quantify the representation of computational biology on English Wikipedia, comparing against several related subfields and Wikipedia overall, using a normalized article quality score defined by Jemielniak *et al.* (2021). Further, we characterize the relationships of computational biology Wikipedia articles with ISCB COSIs by creating a binary COSI-Article matrix linking COSIs to relevant Wikipedia articles. Finally, we apply similarity measures and clustering to characterize relationships between COSIs.

### 2.1 Defining computational biology articles

Wikipedia articles relevant to computational biology were defined as articles tagged as being within the scope of the Computational Biology taskforce of WikiProject Molecular Biology; articles are tagged as such by Wikipedia editors (often WikiProject members) on the articles’ talk pages, which provide a place for discussion of the articles. A list of relevant articles (https://wp1.openzim.org/#/project/Computational_Biology/articles) was generated in July 2021 using the WP 1.0 bot, an automated tool which tracks Wikipedia article assessment data (Zheng *et al.*, 2019). In addition to the article titles, metadata including quality and importance ratings for each article were also extracted.

Wikipedia articles are rated for both quality and importance by Wikipedia editors, according to defined scales; these ratings are also accessible via each article’s talk page. Article quality is rated on an increasing scale through Stub, Start, C, B, Good Article (GA) and Featured Article (FA) classes (Table 1). Articles can only be rated GA or FA through an internal peer review process and are relatively rare, representing 0.5% and 0.1% of all Wikipedia articles, respectively. Article importance (or ‘priority’) is rated similarly, on a scale increasing in importance through Low, Mid, High and Top importance (Table 2). Finally, we manually removed redirect articles captured by the WP 1.0 bot, where they redirected to another Wikipedia article that was also tagged as belonging to the Computational Biology taskforce, so as to avoid duplication.

**Table 1.** English Wikipedia quality assessment criteria

Class	Criteria
Featured Article (FA)	The article has attained featured article status by passing an in-depth examination by impartial reviewers.
Good Article (GA)	The article has attained good article status, having been examined by one or more impartial reviewers.
B	The article is mostly complete and without major problems but requires some further work to reach good article standards.
C	The article is substantial but is still missing important content or contains much irrelevant material. The article should have some references to reliable sources, but may still have significant problems or require substantial cleanup.
Start	An article that is developing but still quite incomplete. It may or may not cite adequate reliable sources.
Stub	A very basic description of the topic. Can be well-written, but may also have significant content issues.

*Note*: Article quality assessment criteria for the most common rating classes. More detailed criteria and criteria for other classes may be found on Wikipedia (https://en.wikipedia.org/wiki/Wikipedia:Content_assessment).

**Table 2.** English Wikipedia importance assessment criteria

Class	Criteria
Top	Subject is a must-have for a print encyclopedia.
High	Subject contributes a depth of knowledge.
Mid	Subject fills in more minor details.
Low	Subject is mainly of specialist interest.

*Note*: Article importance assessment criteria for the most common rating classes. Criteria for other optional classes may be found on Wikipedia (https://en.wikipedia.org/wiki/Wikipedia:Version_1.0_Editorial_Team/Release_Version_Criteria).

### 2.2 Defining articles for related fields

We extracted data for comparisons with other WikiProjects and Wikipedia overall using the WP 1.0 bot in October 2021. As in similar studies (e.g. Jemielniak *et al.*, 2021), we compared only English Wikipedia articles (i.e. pages with encyclopedic content, removing list pages, templates, project pages and media files). For this assessment, we also removed articles which had not been assigned an importance rating. In the case of WikiProject Biography, where importance is rated as ‘Core’ (limited to the top 200 biographies) or ‘Other’, we kept all articles. We also removed a small number of articles with an ‘A class’ quality rating from our assessment, since this assessment class is often omitted by WikiProjects which lack a dedicated assessment team and is therefore unusually underrepresented (0.03% of all Wikipedia articles) and not comparable across all of Wikipedia. For the statistics for Wikipedia overall, where an article was associated with multiple WikiProjects with different quality and importance ratings, the highest ratings were retained, as in similar studies previously.

### 2.3 Normalized article quality score

The normalized article quality scores (*Q*) were computed as:

(1) $$Q=\frac{N_{\text{quality}}}{\sqrt{N_{\text{total}}}},$$

where $N_{\text{quality}}$ is defined as the number of ‘quality articles’ within a WikiProject or taskforce (the sum of the articles in the peer-reviewed GA and FA classes) and $N_{\text{total}}$ is defined as the total number of articles within that WikiProject or taskforce. This metric thus balances the number of high-quality articles with a normalization to remove the effect of larger WikiProjects naturally having more high-quality articles and is bounded between 0 and $N_{\text{total}}/\sqrt{N_{\text{total}}}$; the metric has been characterized by Jemielniak *et al.* (2021) as a variance stabilizing transform, appropriate for comparisons of WikiProject size data.

### 2.4 COSI-Article matrix

To create the COSI-Article matrix, a list of ISCB COSIs was extracted from the ISCB website in July 2021. Each article in the computational biology list generated above was classified as being either relevant or not to each COSI by a group of participants at an online hackathon event organized by the ISCB Student Council and the Computational Biology taskforce of WikiProject Molecular Biology. The hackathon was held during the 17th ISCB Student Council Symposium in July 2021. The majority of the hackathon participants were PhD student members of the ISCB Student Council, although postdoctoral and faculty-level participants also contributed to the classification. Hackathon participants were split into small groups and assigned a randomly sampled subset of articles to classify based on the content of each article. Articles were required to be classified as relevant to at least one existing COSI; otherwise no specific directions were given to hackathon participants. Classifications were entered into a shared Google Sheets spreadsheet and participants were encouraged to discuss the classifications within their groups. Following the hackathon, the classifications of a randomly-sampled subset of articles were reviewed by faculty-level participants. The resulting dataset was a binary matrix, relating ISCB COSIs to Wikipedia articles. The data used in this study are available in the Zenodo repository (doi: 10.5281/zenodo.5814765) (Kilpatrick *et al.*, 2022).

### 2.5 Data mining and analysis

Data were imported into R (v.4.1.2) using the googlesheets4 (v.1.0.0) tidyverse package. Article quality ratings were mapped to integer values (Stub = 1, Start = 2, etc.); article importance ratings were mapped similarly. The trend line and confidence intervals in Figure 2A and B were computed using a generalized additive model with restricted maximum likelihood, using the mgcv R package (v.1.8-38) (Wood, 2017). Significance values for article enrichment were calculated by applying a two-sided Fisher’s exact test to the appropriate 2 × 2 contingency table. COSI similarity scores were computed for each pair of COSIs using Jaccard index, for each pair dividing the number of articles relevant to both COSIs by the number of articles relevant to at least one COSI:

(2) $$J(C_{1},C_{2})=\frac{\left|C_{1}\cap C_{2}\right|}{\left|C_{1}\cup C_{2}\right|}.$$

The Jaccard measure computed here provides a real value between 0 (representing pairs of COSIs with no articles in common) and 1 (representing pairs of COSIs with all articles in common). The resulting pairwise Jaccard scores were subject to hierarchical clustering, computed using Euclidean distance and the complete linkage agglomeration method provided by the hclust function in the stats R package. Heatmaps were produced using the pheatmap R package (v.1.0.12).

## 3 Results

### 3.1 Assessing computational biology representation on Wikipedia

**Fig. 1.**
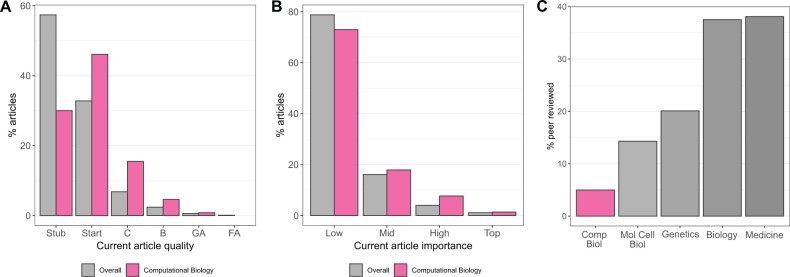
Assessing computational biology representation on Wikipedia. (**A**) Barplot of current article quality ratings, comparing articles identified as relating to computational biology with Wikipedia overall. (**B**) Barplot of current article importance ratings, comparing articles identified as relating to computational biology with Wikipedia overall. (**C**) Proportion of Top importance articles which are in the peer reviewed GA and FA quality classes, for taskforces of WikiProject Molecular Biology (Computational Biology, Molecular and Cell Biology and Genetics) and WikiProjects Biology and Medicine

Our analysis of the data generated by the WP 1.0 bot indicates that computational biology articles have generally higher quality ratings than Wikipedia overall: 8.5% of computational biology articles are rated B or higher (3.5% overall) and 29.9% are rated as the lowest Stub class (55.4% overall) (Fig. 1A). Article importance ratings for computational biology were found to be similar to those of Wikipedia overall (Fig. 1B). Despite a small increase in the proportion of computational biology articles being rated as High or Top importance (9.1%, compared to 5.1% overall), 30.3% of High or Top importance computational biology articles have quality rating B or higher, compared to 21.5% overall. At the other end of the scale, <1% of High or Top importance computational biology articles are rated as Stub class, compared to 14.3% Wikipedia-wide.

However, despite these encouraging statistics, the vast majority of articles (76%) related to computational biology are rated Start class (indicating articles which are either ‘developing but still quite incomplete’ and/or ‘may or may not cite adequate reliable sources’), or lower. Surprisingly, the articles on Bioinformatics and Computational Biology are both rated as Top importance, but remain C class for quality. Furthermore, <1% of computational biology articles are in the peer-reviewed GA or FA classes, with only one Top importance article out of 20, ‘Hidden Markov Model’, amongst these. While this proportion is consistent with the number of GA and FA class articles across Wikipedia, it is notably lower than that of the two other active taskforces of WikiProject Molecular Biology (namely Molecular and Cell Biology, and Genetics) and other related academic WikiProjects such as Biology and Medicine (Fig. 1C).

We note that the Computational Biology taskforce currently has no articles in the highest ‘FA’ quality class. Previously published journal articles which contained summaries of article quality (Bateman *et al.*, 2013; O’Neill *et al.*, 2017) indicated two Mid importance Wikipedia articles in this class; however, further investigation revealed that these articles (on the Folding@home and Rosetta@home protein folding distributed computing projects) were demoted from FA to B Class and C class, respectively, as a result of FA reviews in 2020.

We also found that the normalized quality score (Equation (1)) of the Computational Biology taskforce (*Q *=* *0.32) is also lower than related taskforces and WikiProjects (Table 3). In particular, the Molecular and Cell Biology taskforce of WikiProject Molecular Biology has a higher normalized quality score (*Q *=* *0.50), despite having 20 times as many articles in total. Interestingly, the normalized quality scores of the science-based taskforces and WikiProjects are far lower than that of WikiProject Biography, the largest WikiProject in terms of both the number of articles and number of active editors. For comparison, Table 3 also includes statistics for WikiProject Tropical Cyclones, found in a recent study to be the WikiProject with the highest quality content, despite a relatively narrow scope and small membership (Jemielniak *et al.*, 2021).

**Table 3.** Comparison of WikiProjects on Wikipedia

WikiProject	*Q*	$N_{\text{total}}$	$N_{\text{quality}}$
Molecular Biology/Computational Biology	0.32	1447	12
Molecular Biology/Molecular and Cell Biology	0.50	29 202	86
Molecular Biology/Genetics	0.77	4594	52
Biology	1.13	3338	65
Medicine	1.91	41 637	389
Biography	6.36	1 790 408	8513
Tropical Cyclones	22.50	2834	1198

*Note*: Normalized article quality score (*Q*), number of articles ($N_{\text{total}}$) and number of quality articles ($N_{\text{quality}}$) are given for selected WikiProjects and taskforces.

### 3.2 COSI-Article interactions reveal variability in COSI representation

Our analysis of data from the COSI-Article matrix reveals that computational biology domains have a large amount of variability in their representation on Wikipedia. The Education COSI was found to have the highest number of relevant articles (*n *=* *400), while four COSIs [Critical Assessment of Massive Data Analysis (CAMDA), Microbiome, Text Mining and Junior Principal Investigators (JPI)] were associated with fewer than 100 articles. The CAMDA and Microbiome COSIs both derive from critical assessment challenges, therefore the smaller number of relevant articles may arise from their more specialist nature.

Articles also show wide variability in the number of COSIs that they are identified as being relevant to; the majority of articles are identified as being relevant to three COSIs or fewer, with more than 30% of articles relevant to only one COSI. A small number of general computational biology articles (<1%) are identified as being relevant to 10 or more COSIs, with 4 articles identified as relevant to all COSIs. These articles describe three ISCB-affiliated conferences [ISMB, the European Conference on Computational Biology (ECCB) and the Pacific Symposium on Biocomputing (PSB)] and the ISCB Student Council. Surprisingly, the article on Bioinformatics was not identified as relevant to the JPI COSI and the article on the ISCB was only associated with the BIOINFO-CORE, Education and JPI COSIs.

**Fig. 2.**
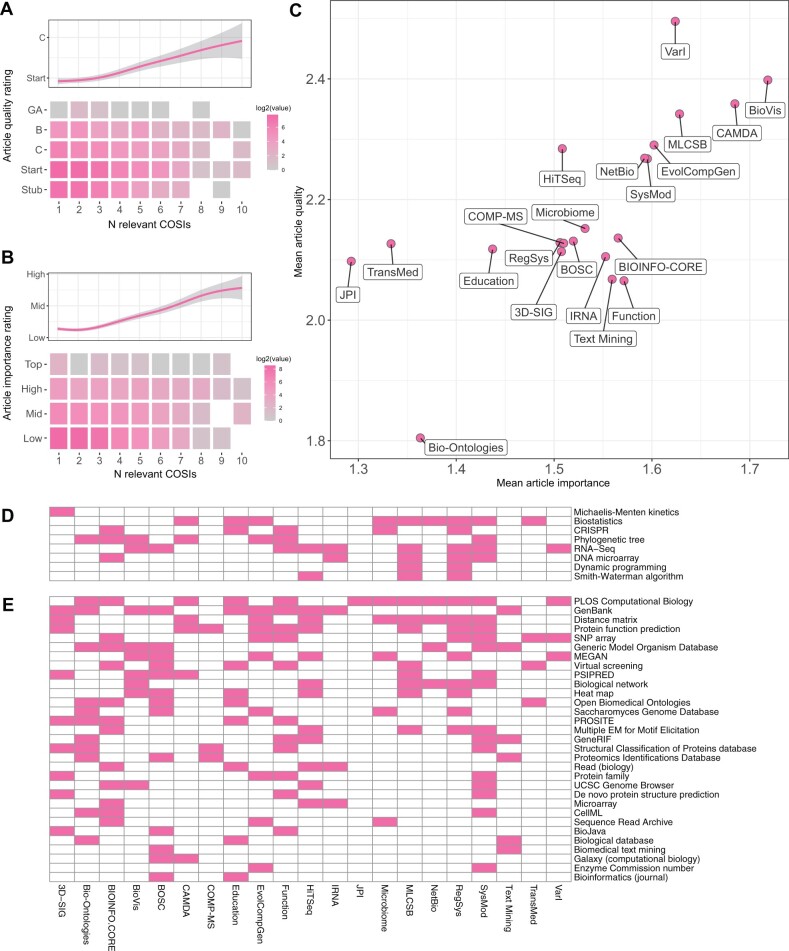
Analysis of COSI-Article interactions. (**A**) **Top:** Trend line with 95% confidence interval for the number of ISCB COSIs associated with each article against the current quality rating. **Bottom:** Heatmap illustrating the number of articles for each combination of number of relevant COSIs and article quality rating. Colors represent the number of articles on a log2 scale; white cells represent no data. (**B**) **Top:** Trend line with 95% confidence interval for the number of ISCB COSIs associated with each article against the current importance rating. **Bottom:** Heatmap illustrating the number of articles for each combination of number of relevant COSIs and article importance rating. Colors represent the number of articles on a log2 scale; white cells represent no data. (**C**) Bubble plot showing COSIs by mean article importance and quality ratings. (**D**) Matrix of Computational Biology taskforce articles which are rated Top importance and B class for quality. Shaded cells indicate relevant COSIs. (**E**) Matrix of Computational Biology taskforce articles which are rated High for importance, Start for quality and are relevant to more than one COSI.

Although many article-level quality and importance ratings were added by members of the Computational biology taskforce of WikiProject Molecular Biology, Wikipedia editors who are not necessarily domain experts may modify these ratings. However, when combined with data from the COSI-Article matrix we find that, as expected, both the current article quality and importance ratings (based on the criteria presented in Tables 1 and 2) display increasing trends as the number of relevant COSIs increases, justifying the use of these metrics in quantifying variability between COSIs (Fig. 2A and B).

Combining data from the COSI-Article matrix with article-level quality and importance metrics allows further comparison of COSI representation on Wikipedia (Fig. 2C). All COSIs were found to have at least one relevant GA class article. We find mean article quality to vary between 1.81 and 2.50 (where 2.0 represents Start class), indicating an opportunity for improved representation across all domains. We find mean article importance to show less COSI-specific variation (1.29 to 1.72); article importance is found to be similar to the Computational Biology taskforce overall.

The Variant Interpretation (VarI) COSI has the highest mean quality (2.50) and highest normalized quality scores (*Q *=* *0.56). These metrics are driven by six GA class articles (the highest among all COSIs), in combination with a relatively small number of articles classed as relevant; the VarI COSI has the highest proportion of GA class articles of all COSIs (5.3%). However, surprisingly, 20.2% VarI articles remain rated Stub for article quality. The Evolution & Comparative Genomics (EvolCompGen) and Regulatory and Systems Genomics (RegSys) COSIs also have 6 GA class articles, but more articles classified as relevant. The normalized quality scores of these COSIs (*Q *=* *0.35 and *Q *=* *0.34, respectively) are higher than that of the Computational Biology taskforce overall.

The Bio-Ontologies COSI is a notable negative outlier in terms of mean article quality (1.80). Despite having three relevant GA class articles, mean article quality is affected by this COSI having the highest proportion of Stub class articles of all COSIs (41.4%). Further analysis reveals that many of these Stub class articles are a brief description of bioinformatics databases which are seldom expanded into more fully-formed articles.

The Biological Data Visualizations (BioVis) COSI scores highly in both mean article quality (2.40) and importance (1.72). However, unlike the above examples, this appears to be principally driven by a lack of low-quality articles: the BioVis COSI has the smallest proportion of Stub class articles of all COSIs (13.9%), significantly lower than the Computational Biology taskforce overall (*P *<* *0.001). Further analysis shows these Stub class articles are all of low importance.

Interestingly, the CAMDA COSI similarly scores highly in both measures (2.36 and 1.68), yet has the lowest normalized quality score of all COSIs (*Q *=* *0.10), suggesting that the articles relevant to this COSI are less likely to be of very good or very poor quality.

Our analysis of data from the COSI-Article matrix reveals a set of eight articles which are currently rated Top importance and B class for article quality (Fig. 2D). These articles are already of higher than average quality and would be good candidates for promotion to GA or FA class via Wikipedia’s internal peer review process. Of these articles, two (Biostatistics and RNA-seq) were classified as relevant to nine COSIs. We also identify a group of 31 ‘high priority’ articles (Fig. 2E): articles relevant to at least one COSI which are currently rated either Top or High for importance and Stub or Start class for article quality. These articles would benefit most from immediate attention from domain experts. Among these, the article on *PLOS Computational Biology* is relevant to most COSIs (*n *=* *12). The Computational Modeling of Biological Systems (SysMod) COSI has the highest number of relevant priority articles (*n *=* *15); however, this is at least partially due to the SysMod COSI having the third highest number of relevant articles among all COSIs (*n *=* *311).

### 3.3 COSI relationships identified by similarity analysis

**Fig. 3.**
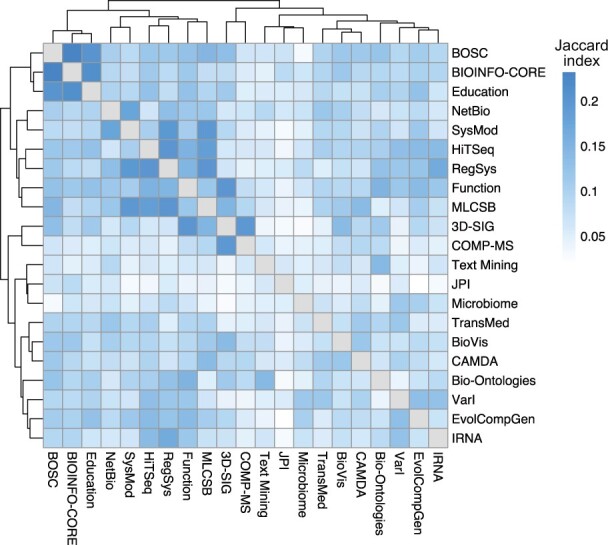
COSI similarity analysis of COSI-Article data. Clustered heatmap of computed pairwise similarities of COSIs. Color represents Jaccard index of each pairwise comparison; higher score represents stronger similarity

We computed pairwise similarities between lists of relevant articles for each COSI using the Jaccard index (Equation (2)). Subsequent hierarchical clustering reveals potential relationships between COSIs (Fig. 3). The highest similarity (*J *=* *0.23) was computed between the Bioinformatics Open Source Conference (BOSC) and BIOINFO-CORE COSIs, at least partially driven by a number of shared Wikipedia articles describing open-source software used in bioinformatics core facilities, including HMMER, MEME Suite, SAMtools and TopHat. These shared articles are also significantly enriched in peer reviewed GA class articles (*P *=* *0.009).

These two COSIs were clustered with the Education COSI and we found high pairwise similarity scores for both BOSC and BIOINFO-CORE with the Education COSI (J=0.20,J=0.21). Almost three quarters of articles relevant to both BOSC and BIOINFO-CORE were also classed as relevant to the Education COSI.

The High Throughput Sequencing Algorithms & Applications (HiTSeq), RegSys, Gene and Protein Function Annotation (Function) and Machine Learning in Computational and Systems Biology (MLCSB) COSIs are clustered together. This clustering is driven in particular by strong pairwise similarity scores between RegSys and both HiTSeq and MLCSB (both *J *=* *0.20). These four COSIs are all well-represented in terms of numbers of relevant articles. Articles relevant to all four COSIs include ChIP sequencing, ENCODE, Protein function prediction and Sequence analysis, suggesting that the strength of this cluster arises from a field which increasingly relies on high-throughput sequencing data analyzed using machine learning techniques. Also among these articles is Transcriptomics technologies, published simultaneously on Wikipedia and in *PLOS Computational Biology* as part of that journal’s Topic Pages initiative (Lowe *et al.*, 2017; Wodak *et al.*, 2012). As expected given the results above we find that, as the number of shared COSIs increases, the average proportion of High and Top importance articles also shows an increasing trend. Surprisingly, 7 out of the 19 articles relevant to all 4 COSIs were rated as low importance, suggesting that the number of relevant COSIs may have a role to play in refining article importance ratings. We find that as the number of relevant COSIs increases, the average article quality also increases; the 19 articles relevant to all four COSIs have the highest average proportion of GA class and only two Stub class articles.

We noted a strong similarity between the Structural Bioinformatics and Computational Biophysics (3D-SIG) and Computational Mass Spectrometry (COMP-MS) COSIs (*J *=* *0.20), driven by articles relevant to protein structure, including the Structural Classification of Proteins database, UniProt, 3D-Jury and the Human Proteome Folding Project.

We also uncovered notable similarities between COSIs from otherwise dissimilar clusters; examples include those between the Bio-Ontologies and Text Mining COSIs (*J *=* *0.14), and the RegSys and Integrative RNA Biology (IRNA) COSIs (*J *=* *0.17). Articles common to the Bio-Ontologies and Text Mining COSIs are mainly descriptions of bioinformatics databases and knowledge resources to which text mining methods may be applied. The small number of articles classed as being relevant to the Text Mining COSI is likely a strong factor: 63% of these were also classed as relevant to the Bio-Ontologies COSI. This is also likely a factor for the latter example, where 46% of articles relevant to the IRNA were also classed as relevant to the RegSys COSI.

The JPI COSI was especially notable for its low similarity to any other COSI (max *J *=* *0.08); we expect this is due to the JPI COSI having the smallest number of relevant articles. Many of the relevant articles for this COSI were biographical, including biographies of many winners of the ISCB Overton Prize, an award honoring investigators in the early to middle phases of their careers (Fogg *et al.*, 2021).

## 4 Discussion

Wikipedia is the most widely accessed OER in computational biology. The collaborative nature of Wikipedia means that, as an educational resource, it has advantages over a standard textbook. We have previously recommended that mentored contributions to Wikipedia from bioinformatics and computational biology students offer opportunities for improving the quality, depth and reliability of publicly accessible knowledge in these fields (Kilpatrick *et al.*, 2020). Similarly, for some years, involvement in WikiProject Medicine has been adopted into the curriculum of some medical schools, with an aim of improving medical students’ ability in communicating with lay audiences (James, 2016). Following our recommendations, computational biology educators at Ohio University (Kilpatrick *et al.*, 2020) and the University of Texas at El Paso (DeBlasio, 2021, 2022) have introduced coursework incorporating the editing of relevant Wikipedia articles. As a result, students have reported improvements in their domain knowledge, scientific writing and other transferable skills, defined as core competencies vital to many bioinformatics roles by the ISCB Education COSI (Mulder *et al.*, 2018).

Open access to scientific knowledge is a core principle shared by the Wikimedia Foundation and the ISCB. Recognizing this, the Computational Biology taskforce of WikiProject Molecular Biology and the ISCB have made continued joint efforts to improve the quality of computational biology Wikipedia articles. These efforts, stretching back more than a decade, have included editathons and workshops at ISCB-affiliated conferences, as well as an annual Wikipedia editing competition (Bateman *et al.*, 2013; Kilpatrick, 2016; O’Neill *et al.*, 2017). As a result, here we find that there is a significantly lower proportion of Stub class computational biology articles than there is in Wikipedia overall (Fig. 1A). However, our comparison to other related scientific fields (Fig. 1C) indicates a need to redouble these efforts and highlights the opportunity for domain experts to make a significant and quantifiable improvement to the quality of computational biology representation on Wikipedia.

The quality measures used in Figure 1C and Table 3 are heavily dependent on the quality of Top importance articles and the number of peer-reviewed GA and FA class articles. The low normalized article quality score for the Computational Biology taskforce is driven by a lack of GA and FA articles; while other WikiProjects have set out goals for promoting articles to GA or FA status (James, 2016), Computational Biology has so far no similar initiative and the total number of these peer-reviewed articles has only increased by two since 2017 (O’Neill *et al.*, 2017). We highlight here a set of eight Top importance articles which are currently rated B class for quality (Fig. 2D). These articles would be excellent candidates for promotion to at least GA status through Wikipedia’s peer-review process; promoting these eight articles to GA status would bring the normalized quality score for the Computational Biology taskforce in line with the Molecular and Cell Biology taskforce. Many other B class articles of lower importance are also candidates for promotion. Improving and promoting these articles to GA or FA status could be a task for graduate students within relevant COSIs. COSI-driven online reviewing and writing sessions focusing on these articles would aid in article improvement and promotion, while fostering communities of junior researchers and their bioinformatics core competencies. We are also actively engaging with COSI leaders and educators to scale-up mentored contributions to Wikipedia from a classroom setting; focusing on article promotion to GA or FA status may form part of these contributions. Further, we identify a set of high-importance but low-quality articles (Fig. 2E), many applicable to multiple domains, which should be a priority for improvement. Beyond these sets of articles, the COSI-level measures of quality we calculated in this study (Fig. 2C) suggest which domains would benefit most from systematic improvement, or creation, of relevant Wikipedia articles.

While article quality and importance ratings are reasonably well-defined, there remains a degree of subjectivity in these ratings, depending on the rating editor. Importance ratings are defined as a perceived expectation that an article’s topic would be covered in a traditional encyclopedia. For a given WikiProject, these ratings are adjusted to consider a reference work in that field. Inevitably, given the diversity of computational biology domains covered by COSIs, an article’s importance will vary between COSIs. However, we find both quality and importance ratings to correlate with the number of COSIs an article was deemed relevant to (Fig. 2A and B), justifying their value as comparative measures.

The clustering of COSIs based on their similarity (Fig. 3) suggests potential synergies between COSIs. On Wikipedia, these could be realized by joint editing events, with the goal of improving articles relevant to both COSIs; quality and importance ratings could be used to prioritize articles for improvement, and cross-COSI discussion could motivate changes to importance ratings. At the community level, these synergies could be fostered by joint conference sessions, or by a shared online seminar series. In a pedagogical setting, it may be useful for educators in the area of one COSI to include examples from other clustered COSIs, to frame their teaching in a wider context. Lists of Wikipedia articles relevant to these clustered COSIs, as defined in this study, may also be used as an educational resource, providing background reading material to supplement the main focus of the class. Encouraging these wider connections would promote links between COSIs, but may also strengthen relationships of members within COSIs.

While the COSI-Article matrix provides powerful insights into computational biology representation on Wikipedia, we acknowledge some limitations of the current dataset. First, there is inevitably some subjectivity in the classification and the potential for unconscious biases in the classification, based on the background of the hackathon participants or the extent of their knowledge of the COSI domains. Second, we expect some variation in domain-level representation depending on the focus of the COSI; for instance, research-based COSIs may expect to have wider representation in an encyclopedia than service-based COSIs (such as JPI or BIOINFO-CORE). Third, the relevance of specific articles to COSIs is naturally subjective and may be overestimated. For example, in assessing the relevance of a random subset of articles to computational biology in general, we found many biographical articles; these may be of lesser relevance to research-based COSIs, but were likely classified as most relevant to research-based COSIs in the absence of a more suitable classification. Many of these biographies were also associated with the Education COSI, which had the highest number of relevant articles. In some cases, the motivation for this classification may be based on the subject’s role as an educator but not necessarily relating to bioinformatics pedagogy. We suggest that many other articles classified as relevant to the Education COSI may similarly be only tangentially related to computational biology education and may be indicative of articles which are less relevant to computational biology in general.

We propose two strategies to ameliorate these limitations. First, involving representatives of all COSIs in the classification and verification processes should reduce biases by increasing participation and having a broader selection of domain experts to verify article relevancy. Increasing the number of postdoctoral and faculty-level participants to balance PhD student participants would also increase expertise to judge article relevancy in these processes. Second, introducing a relevancy scale, extending from binary data to integer data (e.g. on a 0-10 scale) would provide finer-grained information as to article relevance, which is almost certainly different with regard to each different COSI. Given the continually evolving nature of Wikipedia, we expect refining the dataset to be an iterative activity; we propose an annual hackathon event to address this and make other improvements to computational biology representation on Wikipedia. The iterative process for refining bioinformatics core competencies (Mulder *et al.*, 2018; Welch *et al.*, 2014, 2016) within the Education COSI could be a model for dataset refinement, particularly in a group discussion session at in-person events.

There are several other important and related tasks which could be carried out as future hackathons, either as part of the ISCB Student Council Symposium (Hassan *et al.*, 2018) or the main ISMB conference (Gibson, 2012). These tasks may include creating a supervised learning system to refine the COSI-Article classifications, based on keywords supplied by each COSI. Refining the classifications in this way may reduce the subjectivity inherent in the current classification. COSI-defined keywords could also be used to systematically identify existing articles that are relevant to computational biology but not currently tagged as such and are therefore not part of the dataset used in this study. Such a system could be implemented using the Pywikibot library, one of the most commonly used frameworks for creating Wikipedia bots to perform maintenance of MediaWiki sites (Pfundner *et al.*, 2015). Alternatively, a hackathon event may define tasks for a Wikipedia bot to carry out; these could be sent as requests to an existing bot. Another important task would be ensuring that article ratings are up-to-date. Given that computational biology is a fast-moving field, accurate importance ratings will help define which articles are the highest priority for improvement. We found a quarter of Top importance articles were classified as relevant to only one COSI, suggesting refinements should be made to the importance ratings or the classification. A related task could be to design a system which allows for COSI-specific importance ratings, either implemented as part of Wikipedia or as a separate indexing system. Furthermore, we suggest holding extensive editathon events in future to improve article quality; these have been held at ISMB conferences previously but may now potentially be COSI-driven, as suggested above. While this study focuses on English Wikipedia, we recommend carrying out similar evaluations in non-English Wikipedias, which are currently extremely underserved: data obtained via SQL queries to Wikimedia’s Quarry web service (https://quarry.wmcloud.org/) suggests 47% of articles analyzed in this study do not have equivalent articles in any other languages. Further, the language with the largest number of equivalent articles (Arabic) represents only 21% of the articles analyzed in this study. Since Wikipedia may be an even more vital resource for non-English speakers worldwide, an important activity at future hackathon events would be the translation of high importance articles into other languages. This could be done in coordination with the ISCB Student Council’s Regional Student Groups (Shome *et al.*, 2019) and ISCB Affiliate groups, which are actively operating in different regions of the world. These tasks themselves correspond with core competencies defined by the Education COSI, especially competencies J (‘command line and scripting based computing skills’), O (‘effective teamwork to accomplish a common scientific goal’) and N (‘effective communication of bioinformatics… with a range of audiences’) (Mulder *et al.*, 2018).

Finally, as indicated above, a substantial proportion of biographical articles currently fall within the scope of the Computational Biology taskforce of WikiProject Molecular Biology; our analysis shows this may be as high as 15% of articles. All of these scientists are notable figures within the field; however, many of these articles are currently rated Start class for quality. Our analysis indicates a need for a dedicated working group to ensure that the work of these scientists is adequately documented and contextualized within the growing history of the field.

## 5 Conclusion

Wikipedia is a key public engagement platform with immediate impacts on scientific literacy. While some members of the scientific community remain sceptical of Wikipedia, it continues to be a widely used educational resource, and academics have a key role to play in improving Wikipedia (Callis *et al.*, 2009; Shafee *et al.*, 2017a). Our analysis identifies areas where representation of computational biology on Wikipedia could be improved, and also indicates similarities between COSI domains, suggesting synergies which may be leveraged to enhance relationships between computational biology communities. Finally, we suggest future directions to address limitations in the current study and further improve a vital source of publicly accessible computational biology knowledge.
